# An examination of racial differences in 5‐year survival of cervical cancer among African American and white American women in the southeastern US from 1985 to 2010

**DOI:** 10.1002/cam4.765

**Published:** 2016-05-17

**Authors:** Janaka Weragoda, Andres Azuero, Suguna Badiga, Walter C. Bell, Roland Matthews, Chandrika Piyathilake

**Affiliations:** ^1^ University of Alabama at BirminghamDepartment of Nutrition SciencesBirminghamAlabama; ^2^ University of Alabama at BirminghamDepartment of FamilyCommunity & Health SystemsBirminghamAlabama; ^3^ University of Alabama at BirminghamDepartment of PathologyBirminghamAlabama; ^4^ Morehouse School of MedicineDepartment of Obstetrics and GynecologyAtlantaGeorgia

**Keywords:** Cervical cancer, racial disparities, survival, survival analysis

## Abstract

Disparities in Cervical Cancer (CC) mortality outcomes between African American (AA) and White women have been studied for decades. However, conclusions about the effect of race on CC survival differ across studies. This study assessed differences in CC survival between AA and White women diagnosed between 1985 and 2010 and treated at two major hospitals in the southeastern US. The study sample included 925 AA and 1192 White women diagnosed with cervical adenocarcinoma, adenosquamous cell carcinoma, or squamous cell carcinoma. Propensity score adjustment and matching were employed to compare 5‐year survival between the two racial groups. Crude comparisons suggested relevant racial differences in survival. However, the racial differences became of small magnitude after propensity‐score adjustment and in matched analyses. Nonlinear models identified age at diagnosis, cancer stage, mode of treatment, and histological subtype as the most salient characteristics predicting 5‐year survival of CC, yet these characteristics were also associated with race. Crude racial differences in survival might be partly explained by underlying differences in the characteristics of racial groups, such as age at diagnosis, histological subtype, cancer stage, and the mode of treatment. The study results highlight the need to improve access to early screening and treatment opportunities for AA women to improve posttreatment survival from CC.

## Introduction

Cervical cancer (CC) is the fourth most common malignant neoplasm in women and seventh overall globally. The global annual incidence was estimated at 528,000 cases, and the annual number of CC‐related deaths was estimated at 266,000 1, 2. In the United States, CC is the third most common genital tract malignancy and approximately 12,042 new cases and 4074 deaths were attributed to this disease in 2012 2, 3. In the past 40 years, as a result of increased surveillance and improved treatment 2, 3, both the incidence and mortality of CC have significantly decreased. Although the decline in mortality from CC has occurred across all racial and ethnic groups, a disproportionate burden of CC has been shown between African American (AA) and White women 4, 5. It has also been found that African American women were more likely to be diagnosed with advanced stage of CC compared to White women 6.

Several studies have found the survival of CC to be higher in White women compared to AA women 7, 8, 9, 10, 11, 12. These racial differences have been attributed to many factors: lack of early detection and stage at presentation 8, 13, nonadherence to recommended follow‐up care of cervical dysplasia 14, and socioeconomic status 15, 16, 17, 18. However, a number of studies have not found racial differences in CC survival after controlling for stage and treatment 19, 20, 21. Given these conflicting results, a number of studies have highlighted a need to further examine the association between CC survival and race 8, 12, 13, 21. Thus, the objective of this study was to assess racial differences in survival from CC among AA and White women using cancer registry data from 1985 to 2010 obtained at two hospitals located in the southeastern US.

## Sample

This study used data (*N* = 2117) from tumor registries at two locations, University of Alabama at Birmingham Hospital (UAB), Birmingham, Alabama (*n* = 1783), and Grady Memorial Hospital (GH), Atlanta, Georgia, (*n* = 334). The registries used ICD‐O‐3 for classification of topography and morphology of CCs. The participants included in the study (*n* = 925 AAs and *n* = 1192 Whites) were diagnosed with any of the most prevalent forms of CC (adenocarcinoma, adenosquamous cell carcinoma, or squamous cell carcinoma) between 1985 and 2010, received treatment (surgery, chemotherapy, or radiation (alone/combined)) at the aforementioned hospitals, had information on demographics (age, race), date of diagnosis, stage of the CC, and at least five years elapsed since diagnosis. Both UAB and GH patients populations were largely consisted of AA and Whites and only a very small proportion was of other demographic groups. Therefore, we excluded those groups from the analysis since meaningful results could not be generated for them. The study was approved by the Institutional Review Boards of the two hospitals.

### Statistical analysis

The purpose of the analysis was to examine differences in 5‐year survival between AA and White women using the combined UAB and GH participant data (*N* = 2117). Because 88% of participants at GH were AA, to examine robustness of results, a separate set of analyses was conducted with the UAB participants only (*n* = 1783). Differences in the proportion of AA and White women by vital status, age, stage of the cancer at diagnosis, mode of treatment, year of diagnosis, location, and histological subtype of CC were determined using descriptive statistics and chi‐squared tests. Because the AA and White groups appeared dissimilar in all characteristics, two strategies were employed to compare 5‐year survival between the two racial groups: propensity‐score adjustment, and matched‐pair analysis. The propensity‐score adjustment proceeded in two steps. First, a conditional inference tree model 22, (a nonlinear recursive partitioning model) was used to estimate the propensity score, that is, the probability of being AA given the covariates (year of diagnosis, age at diagnosis, stage of cancer, mode of treatment, and location). This modeling approach conducts predictor selection and partitioning of the predictor space via statistical tests (i.e., permutation tests, a class of nonparametric tests), while applying control for multiple testing using Bonferroni adjustments to prevent over‐fitting. Tree models can be shown graphically, and can be used for a variety of outcomes, including continuous, categorical, and time‐to‐event. Correct specification of the propensity scores was determined with an analysis of residuals. The next step consisted of using a Cox proportional hazard model to summarize the racial difference in instantaneous risk of death (i.e., hazard), in the form of a hazard ratio adjusted for the propensity score. Deviations from the proportional hazards assumption were examined using time by predictor interactions. The matched‐pair analysis proceeded in two steps. First, for each AA patient, a White match was found using the Mahalanobis distance as multivariate similarity measure, and the GenMatch search algorithm 23 with replacement (i.e., a White patient could be matched with more than one AA patient). Matching with replacement was used because it results in the highest degree of balance in the covariates and the lowest conditional bias 24. Balance in covariates between the resulting matched groups was checked using cross tabulations and effect sizes. The second step consisted of using a frailty model 25 to summarize differences in instantaneous risk of death between the matched groups. This model was stratified by matched‐pair, and included a random effect for participant to account for dependencies among participants in a matched pair, and having some White participants repeated by matching multiple AAs. Deviations from the proportional hazards assumption were examined graphically. A False Discovery Rate (FDR) adjustment was used to control for multiple testing of the same outcome 26. The FDR rate was held at the 5% level. In order to avoid confusing statistical ‘significance’ with clinical importance or consequence (i.e., the large sample size fallacy 27, 28), regardless of significance tests, model‐estimated racial differences in percent survival at 60 months after diagnosis were computed, and hazard ratios of at least 1.57 in magnitude were interpreted as relevant effects 29. This threshold for a hazard ratio is equivalent to a between‐group standardized mean difference of 0.35 (a small to medium effect size)^30^ in the natural log of the survival time (a variance stabilizing transformation)^31^, under exponentially distributed survival time (a parametric survival model 32 with constant and proportional hazards). A final survival tree model was fitted with all the data to examine the strongest predictors of survival under that modeling approach. The analyses were conducted using SAS Version 9.4 (SAS Institute, Cary, NC), and R version 3.2.0 (R Foundation for Statistical Computing, Vienna, Austria). The R packages *party* 33 and *MatchIt* 34 were used to conduct tree modeling and matching, respectively.

## Results

Table 1 shows the characteristics of the sample by race. 44% of the sample was AA. There were statistically significant differences between AA and White women in all characteristics. Higher percentages of AA women were in the older age groups compared to White women. A higher percentage of AA women underwent radiation, chemotherapy, or both as the mode of treatment compared to White women. About 88% of patients from GH were AA compared to 35% at UAB. Table 2 shows the characteristics of the UAB sample, and provides similar conclusions to those extracted from the total sample (Table 1). Among other differences, AA were older at the time of diagnosis, and a higher percentage underwent radiation, chemotherapy, or both as the mode of treatment compared to White women.

**Table 1:** Demographics, location, year of diagnosis, type and stage of the cervical cancer (CC), mode of treatment, and vital status, by race (*N* = 2117)

Characteristics	Overall*N* = 2117 *n* (%)	AA*n *=* *925*n* (%)	White*n *=* *1192*n* (%)	*P*‐value
Age group in years				
>65–93	320 (15)	174 (19)	146 (12)	<0.0001
>55‐≤65	231 (15)	157 (17)	164 (14)	
>45‐≤55	435 (21)	180 (19)	255 (21)	
>35‐≤45	611 (29)	258 (28)	353 (30)	
>16‐≤35	430 (20)	156 (17)	274 (23)	
Age in years				
Mean (SD)	48 (15)	50 (15)	47 (14)	<0.0001
Median (IQR)	46 (22)	48 (23)	45 (20)	0.0004
Location				
UAB	1783 (84)	632 (68)	1151 (97)	<0.0001
Grady	334 (16)	293 (32)	41 (3)	
Year of diagnosis				
2005–2010	417 (19)	164 (18)	253 (21)	0.0108
2000–2004	504 (24)	204 (22)	300 (25)	
1995–1999	358 (17)	173 (19)	185 (16)	
1990–1994	416 (19)	178 (19)	238 (20)	
1985–1989	422 (20)	206 (22)	216 (18)	
Histological subtype				
Adenocarcinoma	264 (12)	72 (8)	192 (16)	<0.0001
Adenosquamous cell carcinoma	80 (4)	25 (3)	55 (5)	
Squamous cell carcinoma	1773 (84)	828 (89)	945 (79)	
Stage of the CC				
1 or 2	1570 (74)	651 (70)	919 (77)	0.0005
3 or 4	547 (26)	274 (30)	273 (23)	
Mode of Treatment				
Surgery only	914 (43)	315 (34)	599 (50)	<0.0001
Surgery with radiation/chemotherapy/both	321 (15)	135 (15)	186 (16)	
Radiation/chemotherapy/both	882 (42)	475 (51)	407 (34)	
Vital status at 60 months of follow‐up				
Dead	769 (36)	411 (44)	358 (30)	<0.0001
Alive	1097 (52)	449 (49)	648 (54)	
Lost to follow‐up	251 (12)	65 (7)	186 (16)	

AA, African American; SD, overall mean age; IQR, interquartile range; UAB, university of Alabama at Birmingham; Grady, Grady Memorial Hospital, Atlanta Georgia.

**Table 2:** Demographics, location, year of diagnosis, type and stage of the cervical cancer (CC), mode of treatment, and vital status, by race, for UAB patients (*N* = 1783)

Characteristics	Overall*N *=* *1783*n* (%)	AA*n *=* *632*n* (%)	White*n *=* *1151*n* (%)	*P*‐value
Age group in years				
>65–93	273 (15)	130 (21)	143 (12)	<0.0001
>55–≤65	269 (15)	112 (18)	157 (14)	
>45–≤55	366 (21)	120 (19)	246 (21)	
>35–≤45	512 (29)	169 (27)	343 (30)	
>16–≤35	363 (20)	101 (16)	262 (23)	
Age in years				
Mean (SD)	48 (15)	51 (16)	47 (14)	<0.0001
Median (IQR)	46 (22)	49 (24)	45 (19)	<0.0001
Year of diagnosis				
2005–2010	347 (19)	99 (16)	248 (21)	0.0045
2000–2004	438 (25)	144 (23)	294 (25)	
1995–1999	297 (17)	117 (18)	180 (16)	
1990–1994	360 (20)	133 (21)	227 (20)	
1985–1989	341 (19)	139 (22)	202 (18)	
Histological subtype				
Adenocarcinoma	242 (13)	53 (8)	189 (16)	<0.0001
Adenosquamous cell carcinoma	67 (4)	13 (2)	54 (5)	
Squamous cell carcinoma	1474 (83)	566 (90)	908 (79)	
Stage of the CC				
1 or 2	1337 (75)	446 (71)	891 (77)	0.0014
3 or 4	446 (25)	186 (29)	260 (23)	
Mode of Treatment				
Surgery only	784 (44)	204 (32)	580 (50)	<0.0001
Surgery with radiation/chemotherapy/both	277 (16)	97 (15)	180 (16)	
Radiation/chemotherapy/both	722 (40)	331 (53)	391 (34)	
Vital status at 60 months of follow‐up				
Dead	623 (35)	279 (44)	344 (30)	<0.0001
Alive	916 (51)	295 (47)	621 (54)	
Lost to follow‐up	244 (14)	58 (9)	186 (16)	

AA, African American; SD, Overall mean age; IQR, Interquartile range; UAB, University of Alabama at Birmingham.

**Figure 1:**
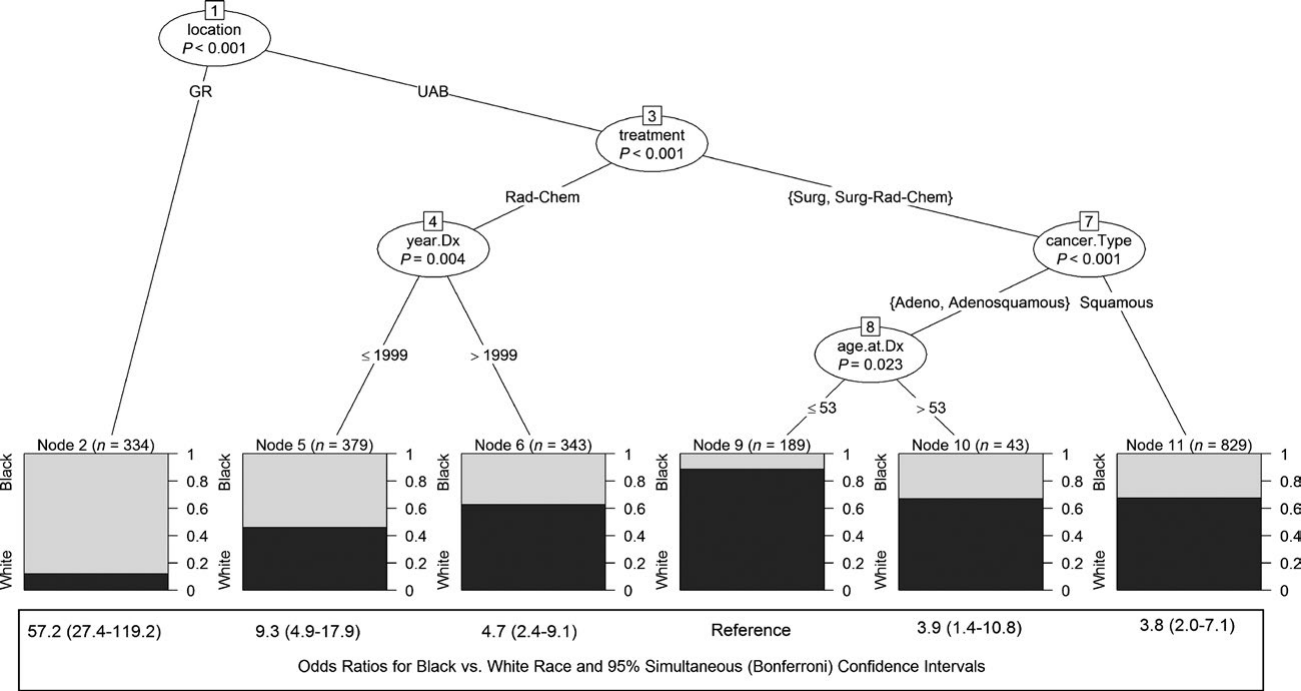
Classification tree model for race, using as predictors location, year of diagnosis, cancer stage, histological cancer subtype, age at diagnosis, and mode of treatment (*N *=* *2117). GR, Grady Hospital; UAB, University of Alabama at Birmingham Hospital; Surg, Surgery only; Rad‐Chem, Radiation or Chemotherapy or both; Surg‐Rad‐Chem, Surgery with Radiation or Chemotherapy or both.

Figure 1 shows the classification tree model predicting race using as explanatory variables: age at diagnosis, year of diagnosis, stage of cancer, histological subtype of cancer, mode of treatment, and location. This model was used to estimate the propensity scores for the analysis that included all participants (*N* = 2117). A residual analysis of the propensity scores showed no relevant residual imbalance in the covariates. A similar modeling approach was conducted using the UAB data only (*n* = 1783). This resulted in the same tree structure with the exception of the split for location (Figure S1). No relevant residual imbalance on the covariates was observed.

The matching algorithm identified 441 White patient matches for the 925 AAs. Since matching was conducted with replacement, some White women matched multiple AA women, and thus the number of pairs was equal to the number of AAs. Of note, since there were only 41 White patients at GH, these patients had to be used repeatedly to match the 293 AAs at that location. The same matching procedure was conducted using the UAB data only, which resulted in 422 White patient matches for the 632 AAs in that location.

As shown in Table 3, no relevant covariate imbalance was found among the matched‐pairs in both the combined locations and using UAB data only.

**Table 3:** Tabulation of characteristics between matched groups overall and at the UAB location

Characteristics	*N *=* *925 Pairs^a^			UAB (*n *=* *632 Pairs)^b^		
	AA	White	Cramer's V^c^	AA	White	Cramer's V^c^
Age group						
>65–93	174 (19%)	129 (14%)	0.08	130 (21%)	122 (19%)	0.02
>55–≤65	157 (17%)	174 (19%)		112 (18%)	119 (19%)	
>45–≤55	180 (19%)	220 (24%)		120 (19%)	119 (19%)	
>35–≤45	258 (28%)	265 (29%)		169 (27%)	168 (27%)	
>16–≤35	156 (17%)	137 (15%)		101 (16%)	104 (16%)	
Year of diagnosis						
2005–2010	164 (18%)	148 (16%)	0.05	99 (16%)	96 (15%)	0.04
2000–2004	204 (22%)	236 (26%)		144 (23%)	156 (25%)	
1995–1999	173 (19%)	151 (16%)		117 (19%)	101 (16%)	
1990–1994	178 (19%)	181 (20%)		133 (21%)	138 (22%)	
1985–1989	206 (22%)	209 (23%)		139 (22%)	141 (22%)	
Histological cancer subtype						
Adenocarcinoma	72 (8%)	74 (8%)	0	53 (8%)	53 (8%)	0
Adenosquamous cell carcinoma	25 (3%)	23 (2%)		13 (2%)	13 (2%)	
Squamous cell carcinoma	828 (90%)	828 (90%)		566 (90%)	566 (90%)	
Stage of cancer						
1 or 2	651 (70%)	652 (70%)	0	446 (71%)	446 (71%)	0
3 or 4	274 (30%)	273 (30%)		186 (29%)	186 (29%)	
Mode of Treatment						
Surgery only	315 (34%)	319 (34%)	0.03	204 (32%)	204 (32%)	0
Surgery with radiation/chemotherapy/both	135 (15%)	153 (17%)		97 (15%)	97 (15%)	
Radiation/chemotherapy/both	475 (51%)	453 (49%)		331 (52%)	331 (52%)	
Location						
UAB	632 (68%)	632 (68%)	0			
Grady	293 (32%)	293 (32%)				

AA, African American; UAB, University of Alabama at Birmingham; Grady, Grady Memorial Hospital, Atlanta Georgia.

a Matching with replacement (*n* = 925 AA and *n* = 441 White).

b Matching with replacement (*n* = 632 AA and *n* = 422 White).

c Cramer's V: measure of association between two nominal variables, giving a value between 0 and 1. Values ~0.1 are considered small, ~0.3 medium, ~0.5 +  large in magnitude.

Table 4 shows the crude, propensity‐score‐adjusted, and matched‐pair estimates of hazard ratios (HR) for race (AA vs. White) using the combined patient data and the UAB location only. Table 4 also shows model‐estimated percent survival differences, and effect sizes that assume exponential survival time. On both the analyses with all participants and UAB‐only, the crude HR of 1.58 crossed the specified relevance threshold (HR>1.57), however, the propensity‐adjusted and matched‐pair HRs did not. All HR estimates were statistically significantly different from the null value of 1 even after correction for multiple testing. Kaplan–Meir curves (for the crude and matched analyses) and adjusted survivor curves (for the propensity‐score‐adjusted analyses) are shown in a Figure S2.

**Table 4:** Crude, propensity score‐adjusted and matched‐analysis estimates of the hazard ratio of death for African American (AA) vs. white women

	Hazard Ratio AA vs. White			Estimated^a^ % survival difference at 60 months	*d* effect size^b^
	Estimate (95% CI)	*P*‐value	FDR		
All participants (*N *=* *2117)					
Crude	1.58 (1.37, 1.82)	<0.0001	0.0007	13.6%	0.36
Covariate adjusted	1.28 (1.01, 1.50)	0.0025	0.0118	7.3%	0.19
Matched‐pair analysis	1.30 (1.09, 1.54)	0.0032	0.0118	8.7%	0.20
UAB‐only (*n *=* *1783)					
Crude	1.58 (1.35, 1.85)	<0.0001	0.0007	13.7%	0.36
Covariate adjusted	1.21 (1.03, 1.43)	0.0167	0.0409	5.3%	0.15
Matched‐pair analysis	1.33 (1.09, 1.65)	0.0063	0.0185	8.9%	0.22

AA, African American; FDR, False Discovery Rate.

Estimated with proportional hazard models.

standardized mean difference in log(survival) time assuming exponentially distributed survival time.

**Figure 2:**
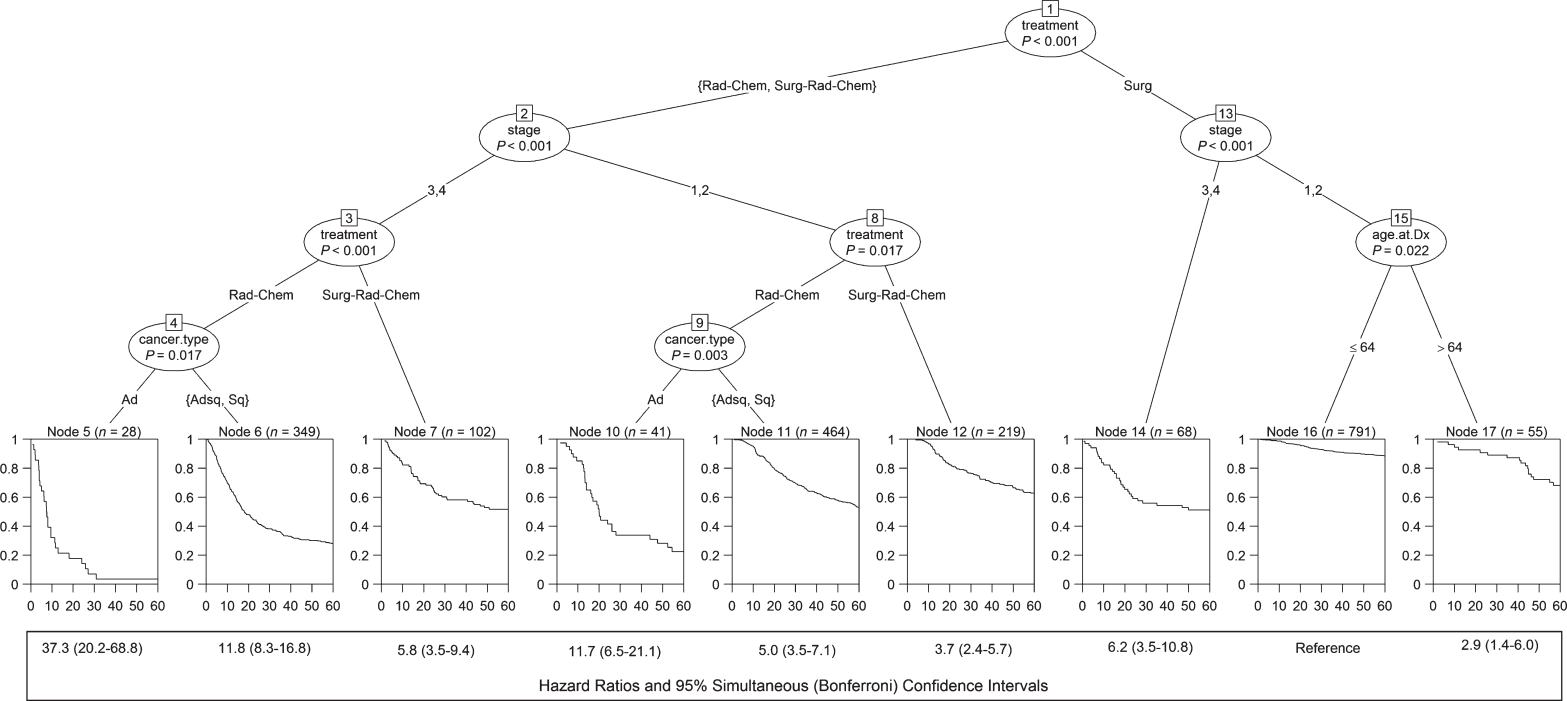
Classification tree model for race, using as predictors year of diagnosis, cancer stage, histological cancer subtype, age at diagnosis, mode of treatment, and excluding location (*N *=* *2117). Surg, Surgery only; Rad‐Chem, Radiation or Chemotherapy or both; Surg‐Rad‐Chem, Surgery with Radiation or Chemotherapy or both.

**Figure 3:**
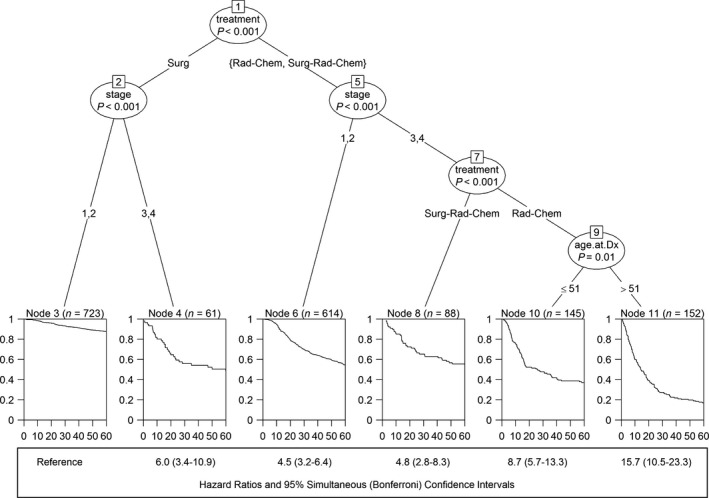
Survival tree model, using as predictors race, location, year of diagnosis, cancer stage, histological cancer subtype, age at diagnosis, and mode of treatment (*N* = 2117). Surg, Surgery only; Rad‐Chem, Radiation or Chemotherapy or both; Surg‐Rad‐Chem, Surgery with Radiation or Chemotherapy or both; Ad, Adenocarcinoma; Adsq, Adenosquamous cell carcinoma; Sq, Squamous cell carcinoma.

Figure 2 shows the survival tree model for all *N* = 2117 participants. The tree algorithm identified mode of treatment, stage of CC, age at diagnosis, and histological subtype as the most salient characteristics predicting survival. The highest survival rate was observed among the *n* = 791 women (terminal node 16) who were 64 years old or younger at the time of diagnosis, had cancer stage 1 or 2, and received surgery only as mode of treatment. The lowest survival rates were observed in three subgroups (terminal nodes 5, 6, and 10) comprising 418 women. Of these, 377 women (nodes 5 and 6) had cancer stage 3 or 4 and their treatment did not include surgery. The 41 women comprising node 10 were diagnosed with adenocarcinoma stage 1 or 2 and their treatment did not include surgery. The smallest HR resulting from this model was 2.9. Figure 3 shows the survival tree model for the *n* = 1783 UAB participants. The UAB‐only model identified mode of treatment, stage of CC, and age at diagnosis as the most salient characteristics predicting survival. The highest survival rate was observed among the *n* = 723 women (terminal node 3) who had cancer stage 1 or 2, and received surgery only as mode of treatment. The lowest survival rates were observed in two subgroups (terminal nodes 10 and 11) comprising 297 patients, who had cancer stage 3 or 4 and their treatment did not include surgery. The smallest HR resulting from this model was 4.5.

## Discussion

This study was conducted to assess racial differences in survival of CC between AA and White women treated in two hospitals in the southeastern US from 1985 to 2010. The crude comparisons suggested relevant racial differences in survival, however, examination of the characteristics (year of diagnosis, age at diagnosis, stage of the CC, mode of treatment, and histological subtype, and location) of the AA and White women indicated that these two groups of women were dissimilar in all characteristics. Comparing ostensibly dissimilar groups remains a challenging aspect of epidemiological studies. Therefore, we used two strategies to attempt to make like‐with‐like comparisons between AA and CA patients. We used propensity score adjustment, and multivariate matching. The multivariable models used to estimate the propensity score were nonlinear recursive partitioning models and therefore allowed detection of complex covariate interactions that would not have been apparent using the more common logistic regression models. Because almost 88% of GH's patients were AA, we conducted analyses with the UAB patients only to determine whether the study conclusions would change based on the inclusion of the GH patients. The conclusions from the propensity‐adjusted and matched analyses concurred in that no large racial differences in survival, as measured by the magnitude of the estimated hazard ratios, were found when attempting to compare like‐with‐like (albeit the estimated small differences were statistically significant). These conclusions were supported by survival regression trees that did not select race as a salient predictor of survival when considered simultaneously with the other available characteristics, and resulted in HRs much higher than those estimated for race (crude or adjusted).

In this study, we found AAs were more likely to be diagnosed at older age than Whites, and the proportion of advanced tumor stage at the time of diagnosis (stages 3 and 4) was higher among AA (30%) compared to White women (23.0%). A greater proportion of AA (66%) underwent both radiation and chemotherapy with or without surgery in compared to Whites (50%). These findings are consistent with published literature 11, 12, 35, 36.

The exploratory survival tree analysis (Figs 2 and 3) did not select race as a salient predictor of survival, given the other set of predictors. Likewise, previous studies do not show consistent results on racial disparity in CC survival. A number of previous studies have reported no racial differences in survival after multivariate adjustment 19, 20, 21, 35, 37 whereas, other studies reported AA women having worse survival compared to white women despite adjustment for several potential confounding variables 7, 8, 11, 12, 13, 18.

A study conducted by Leath et al. 20 using the cancer registry from the UAB Hospital (1994–2000) reported no overall survival difference between White and AA women after adjustment for cancer subtype, stage, and treatment method. However, almost 70% (*n* = 304) of the study sample were White women, and the racial groups were ostensibly dissimilar with regards to demographics and comorbidities, which may have confounding effects on outcome results. In a study of 922 women diagnosed with stages 2 and 3 CCs who were treated with radiotherapy at the Mallinckrodt Institute of Radiology (1959–1993) 19 no racial difference in cancer‐specific mortality was reported. However, the 5‐year overall survival rate for patients who were diagnosed with stage 2 disease was significantly lower in AA women compared with White women (51% vs. 60%). This relatively small difference (OR = 1.44, a small effect size, equivalent to a standardized mean difference of 0.2 in the log odds scale) 38 in survival was attributed to other non‐cancer‐related factors. Brooks et al. 13 evaluated the association of race, co‐morbid illness, insurance status, and other prognostic factors on treatment and survival in 153 women (54% were AA and 46% were White) with invasive CC treated at the University of Maryland and found in the final survival model that AA race (HR 1.9; 95% C.I. = 1.0–3.3) was associated with poor survival. A study conducted with 7237 patients in the Texas cancer registry (1995–2001) by Eggleston et al. 8 found that AA women were more likely to have shorter survival time than non‐Hispanic whites (HR 1.3; 95% C.I. = 1.1–1.5). Regardless of significance testing, this HR is small and its relevance is arguable. Under a simple exponential model, it is equivalent to a standardized difference in log survival time of 0.2 (a small effect size).

In this study, racial differences in survival were not reported by histological subtype due to issues of small counts of White women diagnosed with adenocarcinoma or adenosquamous cell carcinoma at GH. Howell et al. 12 reported significantly higher mortality in patients with adenosquamous compared to those with squamous cell carcinoma. Some studies have reported that histology was not a significant factor for survival 7, 18, 36. In this study, the exploratory tree algorithm that used the entire sample identified mode of treatment, stage of the CC, age at diagnosis, and histological type as the most salient characteristics predicting survival, albeit these variables formed complex interactions, so individual effects for each variable were not summarized. A number of previous studies have also reported age 12, 18, tumor stage 7, 12, 13, 20, mode of treatment 7, 18, 36as independent factors associated with survival. The great majority of studies cited here have used linearized models (Cox proportional hazards models) with direct covariate adjustment as main analytical approach. These models assume proportional hazards, and linearity and additivity of predictors with respect to the log hazard. These assumptions are not always examined, and if they don't hold, the validity of conclusions from such analyses is threatened. Here, we have minimized the use of linearized models to circumvent their limitations. Also, a number of studies used (uncorrected) significance testing as the sole determinant of relevance of a predictor on survival, which in large sample studies opens up the possibility of confusing statistical significance with relevance or consequence. Thus, we have used not only significance tests (appropriately adjusted for multiple testing), but also the magnitude of the effects (i.e., the hazard ratios themselves) to determine whether a difference is likely to be relevant or not, regardless of significance. We have used a threshold of 1.57 for the hazard ratio, equivalent to a standardized mean difference of 0.35 (a small to medium effect size), under some simplifying modeling assumptions. Because choosing a cutoff for an effect size is a subjective decision, we have also presented model‐estimated racial differences in percent survival at 60‐month post diagnosis (Table 4), allowing readers to better judge the magnitude of the observed racial differences in survival. The alternative analytical approaches might explain some of the differences between the results shown here and those in previous studies.

The effect of CC screening and early detection on survival has been highlighted by several studies. Lack of facilities to screen with a Pap test is the most common attributable factor in the development of invasive CC 39, 40, 41, 42. In this study, diagnosis of AAs at an older age and advanced tumor stage compared to White Americans may be due to lower socioeconomic status, access to screening facilities or cultural barriers. However, those factors were not taken into consideration in the analysis because those data are unavailable in the tumor registry databases.

Hicks et al. reported that there is clearly a notable disparity in CC survival between various minority populations and White women. Identifiable factors that affect survival disparity were deficiencies in screening and/or treatment, inappropriate treatment or comorbid illnesses 41. According to the National Health Survey (2010), similar CC screening rates were reported among AA and White Americans 6. However, AA women were more likely to be diagnosed with advanced stage of the disease and the 5‐year relative survival rate for CC is lower compared to the White women. The racial difference in stage at diagnosis may be due to differences in the quality of screening and follow‐up after abnormal pap results 6. Access to quality health care is often compromised among under‐served minorities, particularly AA women, the uninsured and older women 41, 42.

There are several limitations in this study. Important patient characteristics such as socioeconomic status, smoking, alcohol, or drug use, parity, life‐time number of sexual partners, human papillomavirus genotypes present in the CC, other medical comorbidities, access to healthcare/screening facilities, health insurance status, and cultural influence or distrust, refusal or acceptance of treatment were not available for analysis. Although we have included potential confounders in our analyses, residual confounding may still bias our estimates.

## Conclusion

The AA and White women included in the study appeared ostensibly dissimilar in several characteristics. Robust methodology was used in the analyses and the results indicated small racial differences in 5‐year CC survival between AA and White women when attempting to compare like‐with‐like, given the available covariates. Age at diagnosis, stage of the CC, mode of treatment, and histological subtype were identified as the most salient characteristics predicting 5‐year survival, and the race groups differed in these characteristics. Thus, crude racial differences in survival might be partly explained by underlying differences in the characteristics of racial groups, such as age at diagnosis, histological subtype, stage, and mode of treatment. These results highlight the need to improve access to early screening and treatment opportunities for AA women to improve posttreatment survival of CC.

## Conflict of Interest

The Authors made no Conflict of interest.

## Supporting information

Figure S1. classification tree model for race among patients from UAB‐only, using as predictors year of diagnosis, cancer stage, histological cancer subtype, age at diagnosis, and mode of treatment (*n* = 1783).

Figure S2. Kaplan–Meier curves and adjusted survivor curves by analysis type, with FDR‐corrected confidence bands.
