# Supplementary figures and images for: An examination of racial differences in 5‐year survival of cervical cancer among African American and white American women in the southeastern US from 1985 to 2010
Source: Cancer Med. 2016 May 17;5(8):2126–35. doi: 10.1002/cam4.765 (PMC4873605; doi:10.1002/cam4.765)

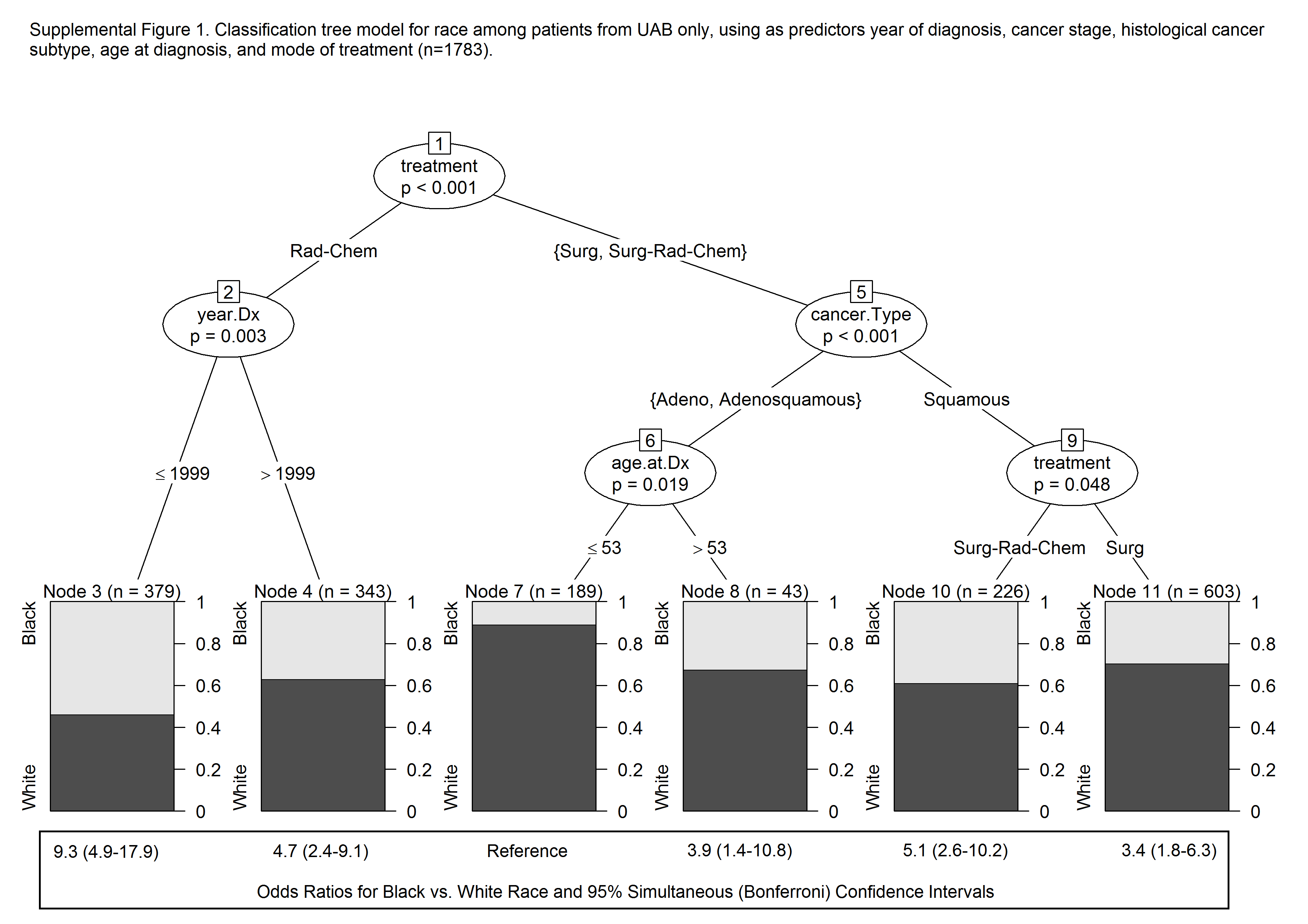

Supplement: Supplementary file 1 — Figure S1. classification tree model for race among patients from UAB‐only, using as predictors year of diagnosis, cancer stage, histological cancer subtype, age at diagnosis, and mode of treatment (n = 1783). [file CAM4-5-2126-s001.tif]

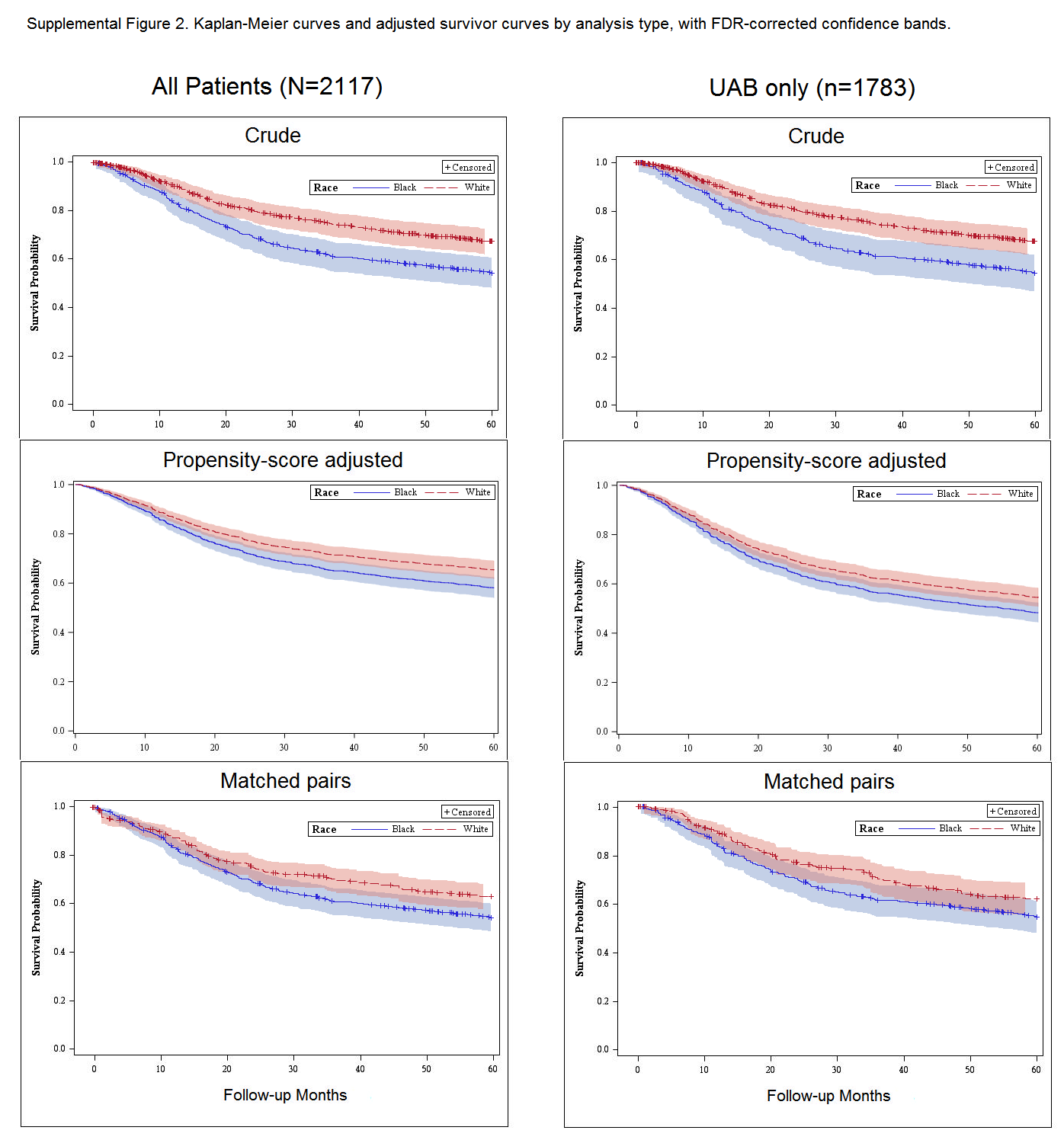

Supplement: Supplementary file 2 — Figure S2. Kaplan–Meier curves and adjusted survivor curves by analysis type, with FDR‐corrected confidence bands. [file CAM4-5-2126-s002.tif]
